# Successful Treatment of Primary Mediastinal Seminoma With Radiotherapy Following Chemotherapy Under a Bloodless Treatment Policy Based on Patient's Religious Sentiments

**DOI:** 10.1002/iju5.70215

**Published:** 2026-07-13

**Authors:** Eisuke Fujii, Yoshiyuki Yamamoto, Kentaro Takezawa, Koji Hatano, Yoichi Kakuta, Atsunari Kawashima, Norio Nonomura, Taigo Kato

**Affiliations:** ^1^ Department of Urology The University of Osaka, Graduate School of Medicine Suita Osaka Japan

**Keywords:** chemotherapy, extragonadal germ cell tumor, Jehovah's witness, mediastinal seminoma, radiotherapy

## Abstract

**Introduction:**

Primary mediastinal seminoma is rare with a favorable prognosis when appropriately treated, and surgical resection after chemotherapy is the standard treatment. However, refusal of blood transfusion for religious reasons can limit curative surgical management.

**Case Presentation:**

A 47‐year‐old Jehovah's Witness man was found to have a 10.8‐cm anterior mediastinal tumor. Biopsy confirmed seminoma. As the patient refused transfusion, definitive chemotherapy followed by radiotherapy was selected. Three cycles of chemotherapy (etoposide, ifosfamide, and cisplatin) resulted in 44% tumor reduction and partial response. Intensity‐modulated radiotherapy of the residual tumor (36 Gy in 20 fractions) achieved complete response, maintained for 11 months without recurrence or late toxicity.

**Conclusion:**

Sequential chemotherapy followed by consolidative radiotherapy appeared to be a safe and effective option for primary mediastinal germ cell tumor in the short term when surgical resection was not feasible for religious reasons.

## Keynote Message

Primary mediastinal seminoma is a rare malignancy with a favorable prognosis. Surgical resection after chemotherapy is standard. When not feasible due to refusal of blood transfusion, consolidative radiotherapy may be a reasonable option and resulted in complete remission in the short term.

**Abbreviations**
BEP: bleomycin, etoposide, cisplatin
CR: complete response
CT: computed tomography
EGGCT: extragonadal germ cell tumor
GCT: germ cell tumor
IGCCCG: International Germ Cell Cancer Collaborative Group
IMRT: intensity‐modulated radiotherapy
OS: overall survival
PFS: progression‐free survival
PR: partial response
VIP: etoposide, ifosfamide, cisplatin

## 1 Introduction

Most germ cell tumors (GCTs) originate in the testes; however, approximately 4%–5% of all GCTs arise at extragonadal sites and represent a rare clinical entity [1, 2]. In adults, the most common primary site of extragonadal germ cell tumors (EGGCTs) is the mediastinum (50%–70%), followed by the retroperitoneum [1, 2, 3]. Non‐seminomatous tumors account for the majority of mediastinal germ cell tumors [1, 2, 3, 4]. Recent updates of the International Germ Cell Cancer Collaborative Group (IGCCCG) classification for seminoma have demonstrated improved outcomes: good prognosis, 5‐year progression‐free survival (PFS) of 89%, 5‐year overall survival (OS) of 95%, and intermediate prognosis: 5‐year PFS of 79% and 5‐year OS of 88% [5]. Overall, a 5‐year OS exceeding 88%–90% is expected for mediastinal seminomas [3, 4, 6]. The standard treatment for primary mediastinal seminoma follows that for testicular tumors and consists of cisplatin‐based combination chemotherapy, such as BEP (bleomycin, etoposide, cisplatin) and VIP (etoposide, ifosfamide, and cisplatin) [4, 6]. However, when a residual mass of > 3 cm remains after chemotherapy, resection of the residual tumor is generally recommended [7].

Such surgical intervention carries a high risk of requiring a blood transfusion, posing a major challenge for patients who refuse allogeneic blood transfusions due to religious sentiments, such as those pertaining to Jehovah's Witnesses, thereby making standard treatment difficult. Herein, we report a case of a primary mediastinal seminoma in a patient who refused a blood transfusion for religious reasons and voluntarily rejected a standard surgical intervention.

## 2 Case Report

**FIGURE 1:**
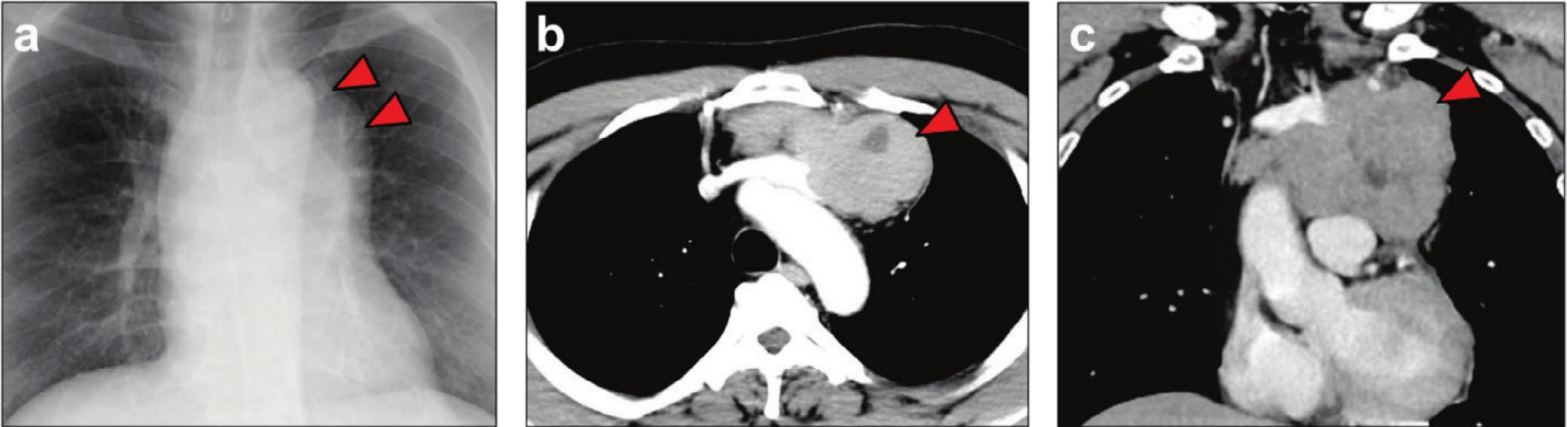
Imaging findings at diagnosis. (a) Chest radiograph. (b, c) Contrast‐enhanced chest computed tomography. (b) Axial images and (c) coronal images. Arrowheads indicate the tumor.

A 47‐year‐old man with no significant medical or family history was referred to our hospital. He had no smoking history and no comorbidities, including hypertension, dyslipidemia, diabetes mellitus, interstitial lung disease, or chronic obstructive pulmonary disease. He was a Jehovah's witness and refused a blood transfusion for religious reasons. Routine occupational chest radiography revealed mediastinal widening (Figure 1a), prompting further evaluation at another hospital. Biopsy confirmed the diagnosis of a seminoma. Due to his strong preference for a transfusion‐free treatment, he was referred to our hospital 2 months after diagnosis. Physical examination revealed no scrotal swelling or palpable testicular masses, suggestive of a mediastinal primary tumor. Tumor markers, including lactate dehydrogenase (231 U/L), alpha‐fetoprotein (4 ng/mL), and human chorionic gonadotropin (< 1 mIU/mL), were within normal ranges. Contrast‐enhanced chest computed tomography (CT) at presentation revealed a well‐defined 10.8‐cm anterior mediastinal mass at maximum diameter (Figure 1b,c). Based on these findings, the patient was diagnosed with a primary mediastinal seminoma, classified as IGCCCG, with a good prognosis.

**FIGURE 2:**
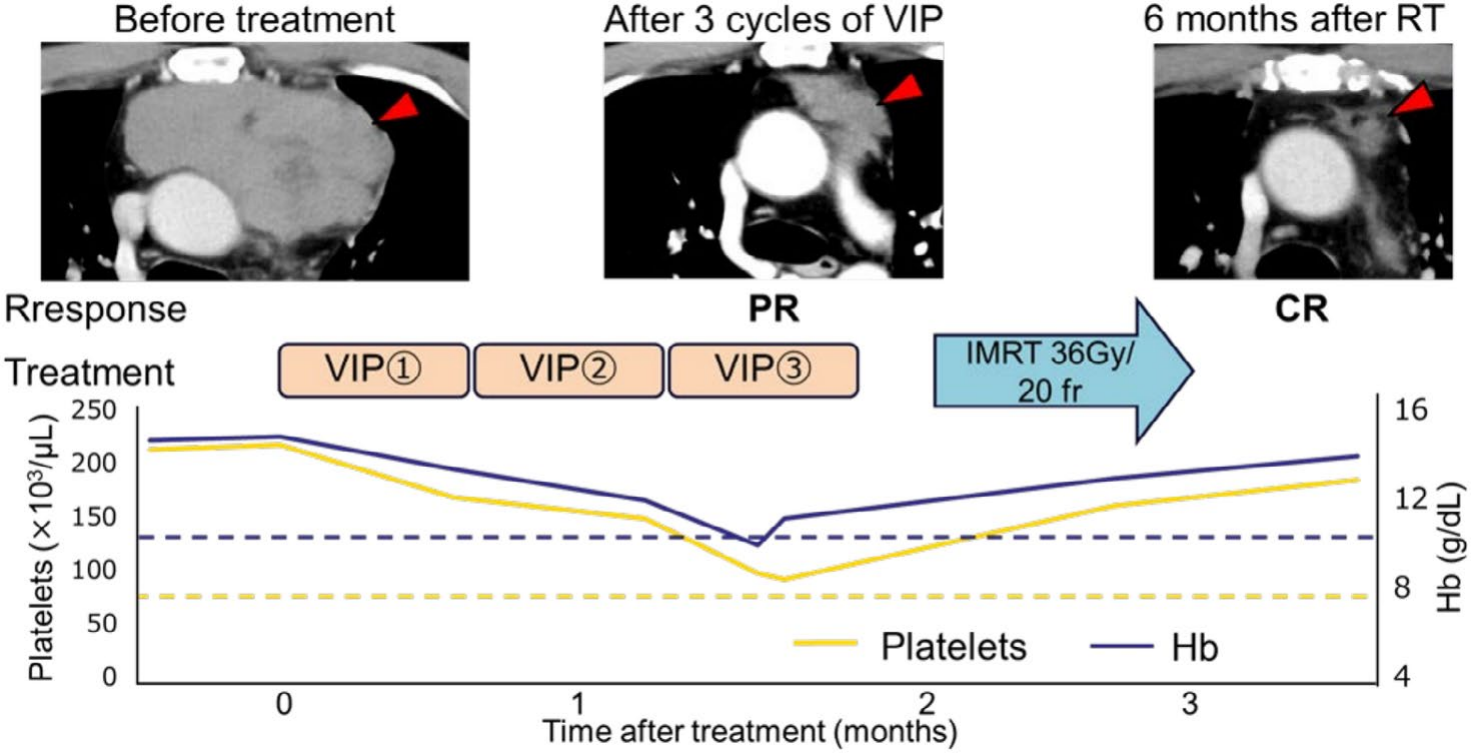
Treatment course. Computed tomography images were obtained at the largest cross‐sectional slice for the pre‐treatment and post‐VIP cycle evaluations, and at the same anatomical level for the assessment at 6 months after radiotherapy. The dashed line indicates a Grade 1 decrease. Arrowheads indicate the tumor. CR, complete response; Hb, hemoglobin; IMRT, intensity‐modulated radiotherapy; PR, partial response; RT, Radiation therapy; VIP, Etoposide + Ifosfamide + Cisplatin.

Standard management recommends surgical resection if a residual mass ≥ 3 cm remains after induction chemotherapy. However, due to the patient's refusal of transfusion and high likelihood of intraoperative blood loss, surgical resection was considered impractical. Detailed considerations are provided in the Supporting Information S1. Therefore, chemotherapy followed by radiotherapy was adopted. Two months after diagnosis, VIP chemotherapy was initiated (etoposide 75 mg/m^2^, Days 1–5; ifosfamide 1.2 g/m^2^, Days 1–5; cisplatin 20 mg/m^2^, Days 1–5). Filgrastim was administered as required for neutropenia, and three cycles were completed. During the third cycle, the patient developed grade 1 anemia (hemoglobin 11.5 g/dL) and thrombocytopenia (138 000/μL), both resolved spontaneously (Figure 2).

Post‐chemotherapy CT demonstrated tumor shrinkage from 10.8 to 6.0 cm (44% reduction), corresponding to partial response (PR). As planned, intensity‐modulated radiotherapy (IMRT) was delivered to the residual tumor 2 months after chemotherapy initiation at a total dose of 36 Gy in 20 fractions. No significant hematological or other adverse events were observed during RT. Six months after radiotherapy, CT revealed marked regression of the mediastinal mass to a scar‐like fibrotic lesion, and the patient achieved complete response (CR). Tumor markers remained within normal limits throughout. Eleven months after IMRT, the patient remained disease‐free.

## 3 Discussion

EGGCTs are rare tumors with a median age of 26 years and a strong male predominance (96.3%) [4]. Histologically, seminoma comprises 34%–48%, while non‐seminomas account for 52%–66% [1, 4]. BEP is the most commonly used regimen (60.5%), followed by VIP (27.8%), yielding CR and PR in 25.3% and 54.4% of patients, respectively [4]. For mediastinal seminoma, 5‐year OS rates are 95% for IGCCCG good prognosis and 88% for intermediate prognosis [5]. Analysis of the Surveillance, Epidemiology, and End Results (SEER) database reported that 3‐ and 5‐year OS rates of 100% in 14 cases of mediastinal seminoma were significantly better than those of mediastinal non‐seminoma [8].

Current guidelines for testicular cancer do not specifically address radiotherapy for mediastinal GCTs [9, 10]. Seminomas are generally considered highly radiosensitive [11]. Retrospective studies from Poland have reported excellent long‐term outcomes with radiotherapy for mediastinal seminoma, with 5‐, 10‐, and 15‐year OS rates of 100%, 91%, and 91%, respectively [12]. Other reports demonstrated durable CR with radiotherapy after chemotherapy [13, 14]. However, bleomycin, a key BEP component, is a known risk factor for radiation‐induced pneumonitis, necessitating caution when planning thoracic irradiation [15]. Furthermore, with regard to mediastinal radiotherapy, careful attention should be paid to potential late adverse events, including radiation‐induced lung injury, cardiovascular complications, and radiation‐associated secondary malignancies [16]. Detailed information is provided in the Supporting Information S1. Despite the heterogeneous reports, the overall outcomes of radiotherapy for mediastinal seminomas appear favorable.

Fluorodeoxyglucose positron emission tomography/computed tomography may be useful for evaluating post‐chemotherapy residual masses in seminomatous germ cell tumors; however, it was not performed in this case. Further discussion is provided in the Supporting Information S1.

From the perspective of refusal of blood transfusion, surgical resection requires careful consideration. Previous studies have reported intraoperative transfusion rates of 67.4% for open thoracotomy and 14% for thoracoscopic surgery for mediastinal tumor resection [17]. For giant mediastinal tumors (> 10 cm), transfusion rates as high as 66.7% have been reported [18]. A recent retrospective study from a German tertiary center reported a transfusion rate of 13% for mediastinal surgery [19]. Given the substantial risk, surgery was excluded from this case.

In 2018, the American College of Surgeons emphasized respect for patient autonomy, individualized assessment of acceptable blood products, and bloodless surgical management for Jehovah's Witnesses [20]. At our institution, surgical treatment is provided in accordance with these guidelines, requiring the submission of a written certificate of transfusion refusal and liability exemption.

This report has some limitations. First, sampling bias from biopsy‐based diagnosis is possible. Although the tumor was diagnosed as seminoma on biopsy and the clinical course was consistent with seminomatous disease, an unsampled non‐seminomatous component cannot be excluded. This distinction is clinically relevant because radiosensitivity and post‐chemotherapy management differ between pure seminoma and mixed germ cell tumors. Another limitation is the short follow‐up of 11 months, which precludes adequate evaluation of long‐term oncological outcomes and late adverse effects.

In conclusion, we report a case of primary mediastinal seminoma in which curative surgical resection was not feasible due to refusal of blood transfusion for religious reasons. Complete remission was achieved with sequential chemotherapy and radiotherapy. This approach may be a safe and effective option in selected patients with similar clinical and ethical backgrounds, although further cases and longer follow‐up are needed.

## Consent

Written informed consent was obtained from the patients for the publication of this case report and the accompanying images.

## Conflicts of Interest

The authors declare no conflicts of interest.

## Supporting information


**Data S1:** iju570215‐sup‐0001‐Supinfo 1.docx.

## Data Availability

The authors have nothing to report.
