# Supplementary material for: Successful Treatment of Primary Mediastinal Seminoma With Radiotherapy Following Chemotherapy Under a Bloodless Treatment Policy Based on Patient's Religious Sentiments
Source: IJU Case Rep. 2026 Jul 13;9(4):e70215. doi: 10.1002/iju5.70215 (PMC13364509; doi:10.1002/iju5.70215)
Supplement: Supplementary file 1 — Data S1: iju570215‐sup‐0001‐Supinfo 1.docx. [file IJU5-9-e70215-s001.docx]

**Supplementary Information**

**Process of treatment decision-making**

Alternative management strategies in this setting could include surveillance after chemotherapy or surgical resection with blood-conservation measures. However, in the present case, a residual mediastinal mass persisted after chemotherapy, and the presence of viable tumor could not be excluded based on computed tomography (CT) findings alone. Therefore, surveillance alone was considered to carry a risk of overlooking residual disease. In addition, although minimally invasive surgery and blood-conservation techniques may reduce the need for transfusion, it remains difficult to completely eliminate the risk of blood transfusion in mediastinal tumor resection. This is particularly relevant in patients who strongly refuse transfusion, as in the present case, making surgical intervention a less feasible option. Accordingly, consolidative radiotherapy was selected as a curative-intent treatment that balances oncological efficacy and safety.

**Key considerations for mediastinal radiotherapy**

When considering mediastinal irradiation, potential risk factors for late cardiopulmonary toxicity should be carefully evaluated. Pre-existing pulmonary conditions, such as interstitial lung disease and chronic obstructive pulmonary disease, as well as smoking history, have been associated with an increased risk of radiation-induced lung injury. ^(1)^ In addition, cardiovascular risk factors, including hypertension, dyslipidemia, diabetes mellitus, and smoking, are known to exacerbate radiation-induced cardiovascular disease. ^(2)^ Furthermore, smoking has been identified as a major risk factor for secondary lung cancer following thoracic irradiation. ^(3)^ Therefore, these patient-related factors should be taken into account when planning mediastinal radiotherapy.

**The role of FDG-PET/CT in primary mediastinal seminoma**

Fluorodeoxyglucose positron emission tomography/computed tomography (FDG-PET/CT) may be helpful in selected cases for evaluating residual masses after chemotherapy in seminomatous germ cell tumors, particularly when residual viable tumor cannot be excluded on CT alone. Previous studies, including the SEMPET trial, have suggested its utility in post-chemotherapy seminoma residual lesions. ^(4)^ However, because the available evidence is largely based on gonadal seminoma, its role in primary mediastinal seminoma remains less well established.

**References**

[1] Hanania AN, Mainwaring W, Ghebre YT, Hanania NA, Ludwig M. Radiation-Induced Lung Injury: Assessment and Management. *Chest*. 2019; **156**: 150-62.

[2] Belzile-Dugas E, Eisenberg MJ. Radiation-Induced Cardiovascular Disease: Review of an Underrecognized Pathology. *J Am Heart Assoc*. 2021; **10**: e021686.

[3] Chen K, Liu C, Li X et al. Risk and prognosis of secondary lung cancer after radiation therapy for thoracic malignancies. *Clin Respir J*. 2024; **18**: e13760.

[4] De Santis M, Bokemeyer C, Becherer A et al. Predictive impact of 2-18fluoro-2-deoxy-D-glucose positron emission tomography for residual postchemotherapy masses in patients with bulky seminoma. *J Clin Oncol*. 2001; **19**: 3740-4.
